# What the ‘Moonwalk’ Illusion Reveals about the Perception of Relative Depth from Motion

**DOI:** 10.1371/journal.pone.0020951

**Published:** 2011-06-22

**Authors:** Sarah Kromrey, Evgeniy Bart, Jay Hegdé

**Affiliations:** 1 Brain and Behavior Discovery Institute, Georgia Health Sciences University, Augusta, Georgia, United States of America; 2 Vision Discovery Institute, Georgia Health Sciences University, Augusta, Georgia, United States of America; 3 Palo Alto Research Center (PARC), Palo Alto, California, United States of America; 4 Department of Ophthalmology, Georgia Health Sciences University, Augusta, Georgia, United States of America; University of Maribor, Slovenia

## Abstract

When one visual object moves behind another, the object farther from the viewer is progressively occluded and/or disoccluded by the nearer object. For nearly half a century, this dynamic occlusion cue has beenthought to be sufficient by itself for determining the relative depth of the two objects. This view is consistent with the self-evident geometric fact that the surface undergoing dynamic occlusion is always farther from the viewer than the occluding surface. Here we use a contextual manipulation ofa previously known motion illusion, which we refer to as the‘Moonwalk’ illusion, to demonstrate that the visual system cannot determine relative depth from dynamic occlusion alone. Indeed, in the Moonwalk illusion, human observers perceive a relative depth contrary to the dynamic occlusion cue. However, the perception of the expected relative depth is restored by contextual manipulations unrelated to dynamic occlusion. On the other hand, we show that an Ideal Observer can determine using dynamic occlusion alone in the same Moonwalk stimuli, indicating that the dynamic occlusion cue is, in principle, sufficient for determining relative depth. Our results indicate that in order to correctly perceive relative depth from dynamic occlusion, the human brain, unlike the Ideal Observer, needs additionalsegmentation information that delineate the occluder from the occluded object. Thus, neural mechanisms of object segmentation must, in addition to motion mechanisms that extract information about relative depth, play a crucial role in the perception of relative depth from motion.

## Introduction

When one visual object moves behind another, it provides a compelling sense of their relative depth, or depth-order (*i.e*., which object is closer to the viewer and which object is farther in depth). Depth-order from motion (DFM) is one of the strongest and the most ubiquitous cues to depth-order under natural viewing conditions[1], [2], [3], [4], [5], [6], [7], [8], [9]. Indeed, DFM can override depth-order from many other types of depth-order cues, including static occlusion[8], [10]. DFM can also resolve ambiguities from other depth-order cues[8], [10]. The neural mechanisms of DFM are almost entirely unknown. Thus, observations that can constrain and inform the search for the neural correlates of DFM are very valuable.

Previous studies have identified two distinct types of DFM cue (for overviews, see[8], [11], [12], [13], [14]; also see Supporting Information S1 about the biasing effects of motion shear; [15]). For instance, when a window shade is drawn shut, it progressively occludes scene elements outside the window. Conversely, when the shade is pulled open, those same scene elements are progressively disoccluded. This dynamic occlusion/disocclusion has long been recognized as a potential cue for depth-order and has been termed the accretion-deletion (AD) cue, also referred to as the dynamic- or kinetic occlusion cue[1], [2], [3], [4], [6], [7], [8], [9]. This cue is the focus of this study. The other DFM cue, called the boundary flow cue (BF cue, also referred to as the common motion cue) results from the fact that the surface elements of the occluder move coherently with the occlusion boundary (*i.e*., boundary between the occluder and the occluded object)[9], [11], [16].

For the last several decades, it has been thought that the AD cue can elicit the DFM percept by itself, *i.e*., that AD cue is self-sufficient[1], [2], [3], [4], [6], [7], [8], [9]. This would appear geometrically self-evident, since the surface undergoing occlusion/disocclusion is always the farther surface. The self-sufficiency of the AD cue, if valid, has important implications for the neural mechanisms by which the brain extracts DFM information from the AD cue. For instance, the underlying neural processes, and the experimental approach to finding them, will have to be fundamentally different if the brain can determine DFM solely by tracking the accretion-deletion of image elements (*i.e*., if the AD cue is self-sufficient), *vs*. if it has to explicitly determine an additional parameter, such as the border at which accretion-deletion occurs. If depth-order cannot be determined solely from the occlusion/disocclusion of the occluded object, *e.g.*, information about the occlusion border is also needed, it will mean that DFM perception cannot be implemented solely by first-order (*i.e*., luminance-based) motion mechanisms[1], [2], [3], [4], [6], [7], [9]. Moreover, it will mean that the AD cue and the BF cue are not mutually redundant. This will mean, in turn, that the current view of DFM as a process supported by two equivalent cues[1], [2], [3], [4], [6], [7], [9], [16] is not valid. Altogether, the question of whether the AD cue is self-sufficient is crucial to understanding how we perceive depth-order from motion.

In this report, we use Ideal Observer analysis to show that the brain, in principle, could determine depth-order from dynamic occlusion alone, consistent with the longstanding belief. However, usingcontextual manipulations of a previously reported motion illusion ([17], [18]; also see Demo Movies 1 and 2, downloadable from www.hegde.us/DFMdemo1.avi and www.hegde.us/DFMdemo2.avi, respectively), which we refer to as the “Moonwalk Illusion” for convenience, we empirically demonstrate that the human brain cannot determine depth-order in this fashion and that it needs additional information that segments the occluder from the occluded object.

## Materials and Methods

### Subjects

Eleven (6 female) adult volunteer subjects with normal or corrected-to-normal vision participated in this study. All subjects provided written informed consent prior to the study. The institutional review boardof the Georgia Health Sciences University, called the Human Assurance Committee (HAC), specifically approved this study. This investigation was conducted according to the principles expressed in the Declaration of Helsinki.

### Stimuli and Task

**Figure 1:**
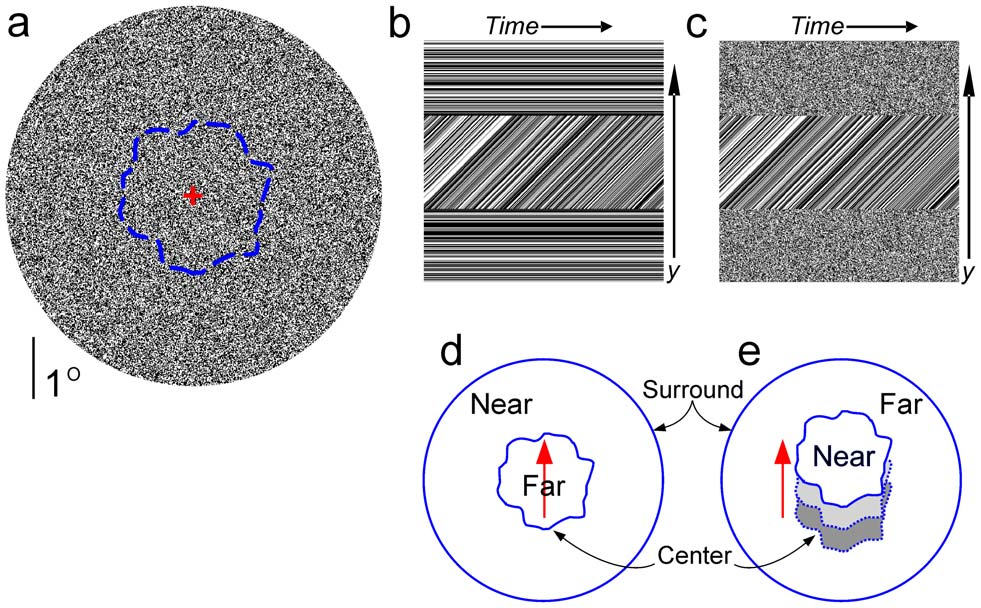
Stimuli and percepts. (a) A single frame of a typical motion stimulus. Unless noted otherwise, each stimulus consisted of two random dot surfaces: a center surface with an irregular outline (imaginary blue line) surrounded by an annulus. Also, the annulus, as well as the outline of the center, remained stationary in all stimuli. Subjects reported the depth-order of the center using a key press. (b,c) Space-time (ST) plots [5], [9] of the two main motion stimuli used in this study (see Supporting Information S1 for the details of ST plot construction). Depending on the condition, the dots of the surround (top and bottom strips) remained static (b) or flickered to various degrees (stimulus in (d) denotes maximal flicker). In all stimuli, the dots of the center translated smoothly (upward in the case shown), and this motion was pixel-to-pixel identical across all stimuli regardless of the surround flicker, as denoted by the fact that the lines in the middle strip are identical between (b) and (c). Also see Demo Movies 2 and 1, downloadable from www.hegde.us/DFMdemo2.avi and www.hegde.us/DFMdemo1.avi, respectively. (d,e) Percepts elicited by the stimuli in (b) and (c), respectively. When the surround dots were static, the center was perceived as a moving far surface visible through a hole in the nearer surround (d). When the surround dots were flickering, the center appeared to be the near surface moving over the farther surround (e). The effect in (e) was previously reported by Anstis and Ramachandran [17], [18]. The effect in (d) is original to this study to our knowledge. Note that the depth-order reversal of the center is entirely a contextual effect, in that it occurs solely as a result of the changes in the flicker of the surround in the total absence of changes in the center.

The experiments were carried out largely as described previously (Hegdé, et al., 2004). Both the center and surround of each stimulus (Fig. 1) consisted of random dot surfaces with a dot density of 50% and, unless noted otherwise, a Michelson contrast of 1.0. The center and the surround did not physically move relative to each other. Unless noted otherwise, the surface properties of the center vs. surround were identical, so that the two surfaces were indistinguishable in any single given frame. To create a surround with a given flicker, we set the probability of a given surround dot surviving one frame to a value between 50% (random flicker) to 100% (static dots). In order to reduce the contrast of a given surface, we reduced contrast of the dots while keeping the mean luminance unchanged.

Stimuli (each 6.2O dia) were presented centered on the fixation spot on a Dell 75 Hz LCD display against a neutral gray background. The center dots all moved coherently at 6O/s in a given direction during a given trial, but the direction of motion varied randomly from trial to trial. Subjects viewed the stimuli for 4 s while maintaining fixation (with blinks as necessary), and reported the depth-order of the center using a key press. Subjects were told to report the depth-order they perceived without regard to what the expected or ‘correct’ depth-order was. No feedback was provided. All trials were randomly interleaved.

### Estimating optic flow

Optic flow was estimated using the algorithm of Horn et al (Horn &Schunck, 1981) using software custom-written in Matlab (Mathworks Inc., Natick, MA).

## Results

### Depth-order Perception in Moonwalk Stimuli

The stimulus consists of two random dot surfaces: A central surface of coherently moving random dots surrounded by an annulus of flickering random dots. Although both the surfaces themselves are stationary, *i.e*., the outline of neither surface actually moves, the central surface (or ‘figure’) appears to translate in the direction of the moving random dots ([17], [18]; see Demo Movie 1, downloadable from www.hegde.us/DFMdemo1.avi). This motion illusion, which we refer to as the Moonwalk illusion, also has a depth-order dimension: The central figure appears to be nearer to the viewer than the flickering surround (see below). This DFM percept is the opposite of that expected from the AD cue arising from the dots of the figure undergoing accretion/deletion at the border between the center and the surround. Therefore, this illusion offers an excellent test case for studying the factors that influence DFM by the AD cue.

Our stimuli consisted of various contextual manipulations of the Moonwalk stimuli that left the accretion/deletion of the center dots completely unaffected. Except where noted otherwise, the stimuli consisted of an irregular central disc of moving random dots surrounded by stationary annulus of similar random dots (Fig. 1).

**Figure 2:**
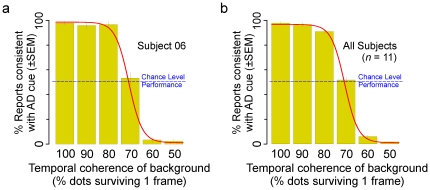
Dependence of the AD cue on contextual information. The temporal coherence (i.e., amount of flicker) of the surround was systematically modulated while the amount of accretion-deletion information was held constant, as described in Materials and Methods. Increasing values of temporal coherence denote decreasing flicker, with 100% temporal coherence denoting static dots. The reported depth-order percepts are shown as the percentage of trials in which the depth-order percept was consistent with the percept predicted by the AD cue by itself, i.e., that the center surface was far. (a) Reported percepts of a representative subject, and (b) average across all subjects. The red line in either panel denotes the best-fitting logistic regression line.

To ascertain that the AD cue in our stimuli was indeed capable of eliciting the DFM percept predicted by the AD cue, we first tested a version of this stimulus in which the surround dots were static (Fig. 1a; also see Demo Movie 2, downloadable from www.hegde.us/DFMdemo2.avi). In this stimulus, the AD cue is the sole depth-order cue, caused by the occlusion-disocclusion of the dots of the moving center by the stationary occluder *i.e*., the surround. Specifically, the BF cue is absent in this stimulus, since this cue arises only when the occluder itself moves[8], [9], [11], [16], whereas the occluder is stationary in this case. Since the texture elements undergoing accretion-deletion belong to the center, the predicted depth-order is that the center is perceived as the far (occluded) surface, and the surround is perceived as the near (occluding) surface. When the surround dots are static (*i.e*., 100% coherent from one frame to the next), this is indeed the reported percept (binomial proportions test, *p*<<0.01; Fig. 2a; also see Supporting Information S1).

We then made this stimulus progressively closer to the original Moonwalk stimulus by introducing flicker to the surround while leaving the center unchanged (Fig. 1b; also see Demo Movie 1, downloadable from www.hegde.us/DFMdemo1.avi). We hypothesized that if the AD cue is sufficient by itself for DFM perception, *i.e*., if the visual system can determine the depth-order solely by measuring the accretion/deletion of the pixels of the center, then manipulations of the surround that leave the center entirely unaffected should leave the DFM percept unaffected. Note that the center in this stimulus was pixel-to-pixel, frame-to-frame identical to the stimulus shown in Fig. 1a, so that the available accretion-deletion information was identical between the two stimuli. If the AD cue were self-sufficient for determining the DFM percept, this stimulusis expected to elicit the same depth-order percept as the stimulus with the static surround.

However, with the flickering surround, the DFM percept reversed, in that the subjects perceived the center as the nearer surface. Moreover, the variations in the flicker accounted for all non-random variation in the DFM percept (logistic regression, *r*2 = 0.83; *p*<0.05; chi-square test for the normality of the residuals, *p*>0.05). Together, these results indicate not only that the AD cue was not sufficient by itself to account for the observed DFM percept, but the AD cue along with the ‘gating’ information in the surround were sufficient.

### AD Cue is Self-Sufficient from the Computational Viewpoint

One potential concern about interpreting the above results as evidence of perceptual insufficiency of the AD cue is that it may not be possible to determine depth-order solely by measuring the accretion and/or deletion of the occluded object to begin with. This would mean that thedefinition of the AD cue as solely a function of the accretion/deletion of the occluded object without reference to the occluder *per se*, although widely accepted in the field [1], [2], [3], [4], [5], [6], [7], [8], [9], may nonetheless be artificially narrow.

To verify that the information from the accretion/deletion of the occluded object is, from a computational point of view,self-sufficient for determining DFM unambiguously, we carried out an Ideal Observer analysis (see Supporting Information S1 for details). We found that the Ideal Observer can indeed determine DFM solely using the accretion/deletion of the center, regardless of the surround. Briefly, the Ideal Observer need only to evaluate the pixels *i* in the image region where a portion of the pixels get deleted over two given successive frames 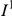 and 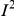 (which we refer to as area 3; see Figure S2 in Supporting Information S1) to determine the log-likelihood ratio
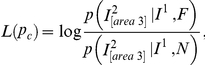



where *F* and *N* are the depth-order models where the center is far or near, respectively, and 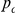 is the parameters of the Ideal Observer model. Namely, 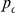 is the probability with which a given center pixel switches (from on to off, or vice versa) from frame 1 to frame 2. The Ideal Observer analysis also shows that the strength of the DFM information is not affected by flicker (see eq. 12 in Supporting Information S1). Thus, purely from an information processing viewpoint, it is possible to extract DFM information from the accretion-deletion information alone.

**Figure 3:**
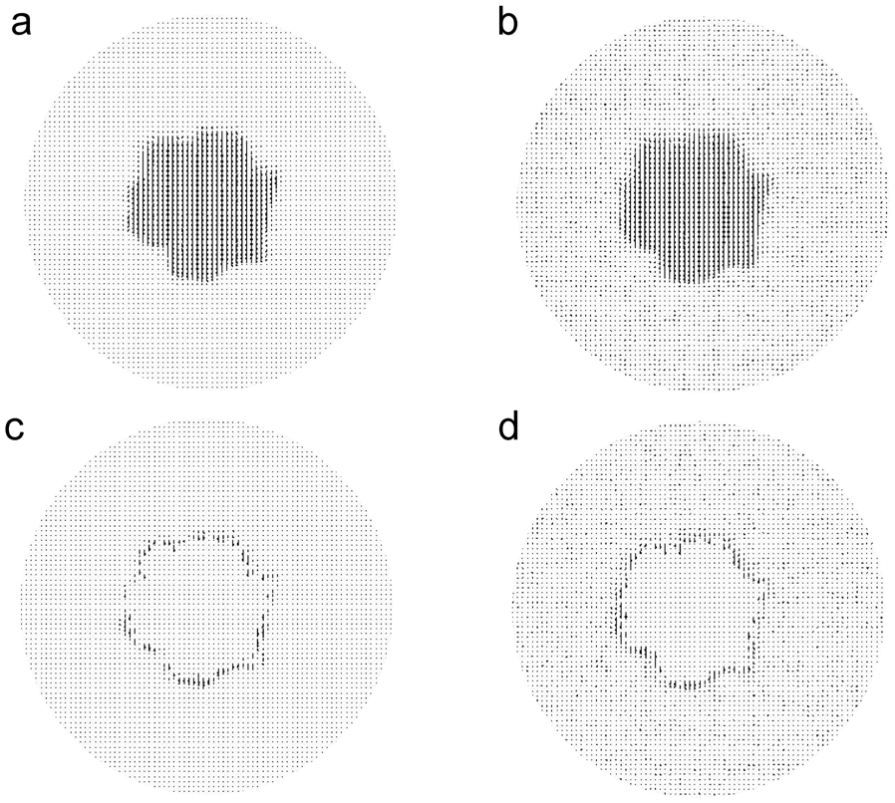
Optic flow information in the center with or without surround flicker. Motion information of our stimuli was estimated using a conventional optical flow estimation algorithm [19]. (a,b) Optical flow field when the surround was static (a) or flickery (b). (c,d) Accretion-deletion zone, or the region of the center where the dots underwent accretion/deletion from one frame to the next, estimated with a static surround (c) or flickery surround (d). Note that the estimated surround flicker has little effect on the estimated optic flow or the estimated accretion-deletion zone of the center itself even with this relatively simple optic flow algorithm, although corresponding estimates of the surround are somewhat different between the two conditions.

We also independently verified, using conventional optic flow algorithms[19] to analyze the two types of motion stimuli shown in Fig. 1, that the changes in the flicker did not affect the optic flow information in the center (Fig. 3a *vs*. b). The sub-region of the center in which the dots underwent accretion/deletion was also readily identifiable regardless of the surround flicker (Fig. 3c *vs*. d; see legend for additional details). Together with the Ideal Observer analysis, these results demonstrate that the available AD information is, in principle, sufficient to support the determination of DFM, even though the visual system is unable to exploit this information to determine DFM. These computational analyses also suggest that our psychophysical results are not a semantic side effect of defining the AD cue too narrowly, *i.e*., separately from the boundary information.

### ‘Gating’ of the AD Cue by Segmentation Information

An inspection of the motion stimulus represented in Fig. 1c (see Demo Movie 1, downloadable from www.hegde.us/DFMdemo1.avi) indicates that when the surround is flickering, it becomes perpetually difficult to delineate the border between the center and surround. This suggests that one reason why the AD cue is ineffective with the flickering surround is that the visual system, unlike the Ideal Observer, needs a mechanism for delineating the occluder in order to make use of the AD cue. In other words, although the visual system cannot determine the depth-order using the AD cue alone, it can do so when the AD cue is augmented by information about the occluder. If this is true, then the DFM percept expected from the AD cue should be restored, notwithstanding the surround flicker, when center-surround segmentation is made easier by an extraneous segmentation cue.

**Figure 4:**
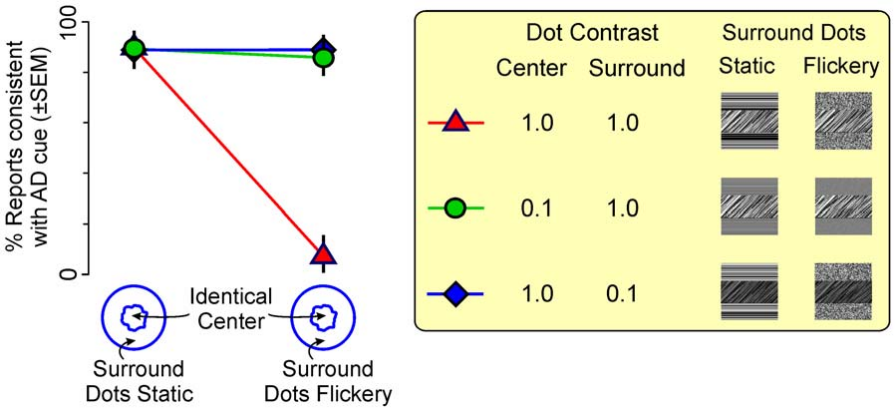
Restoration of the AD percept by the addition of center-surround segmentation cues. Stimuli shown in Fig. 1 were re-tested with or without additional segmentation cues to enhance the delineation of the center vs. surround. Three pairs of conditions were used (inset): with the dots in the surround at a lower contrast (blue diamonds), with the dots in the center at a lower contrast (green circles), or no contrast manipulations (red triangles; condition identical to that in Fig. 1, used as a control). The depth-order percept elicited by each of the conditions is shown as a proportion of reports consistent with the AD cue, i.e., that the center is farther than the surround. Note that the contrast manipulations do not add depth-order or motion information, because the difference in contrast is not a depth-order cue or a motion cue. The surround dots were either static or flickery (100% or 50% temporal coherence, respectively). The flicker of the surround dots was unaffected by the contrast manipulations.

To test this hypothesis, we carried out an additional experiment, where we made the surround more readily distinguishable from the center by changing the luminance contrast of the dots either within the center or within the surround, while leaving the other surface unchanged (Fig. 4, inset). We then measured the perceived depth-order as a function of the flicker of the surround. When the surround dots were static (Fig. 4, left column), this manipulation made no difference; the perceived depth-order was consistent with the AD cue, as in the earlier experiment. When the surround dots were flickery and the dot contrast was the same between the center and the surround (red triangle, right column), the center was perceived as near, also as expected from the earlier experiment. However, when the center-surround segmentation was made easier by lowering the contrast of surround dots while leaving the center unchanged, the depth-order expected from the AD cue was restored, even though the surround flicker was unchanged (green circle at right; binomial proportions test, *p*<0.05). This restoration was not a function of the lower dot contrast in the surround *per se*, because the same restoration occurred when the center-surround distinction was mediated by the lower contrast in the center, instead of in the surround (blue diamond at right; binomial proportions test, *p*<0.05). Note that this restoration of the DFM percept predicted by the AD cue is not attributable to the contrast manipulations *per se*, since this provides no depth-order information whatsoever. Moreover, the same effect was obtained when color or luminance, instead of contrast, was used as the segmentation cue (data not shown).

The fact that the effect of the AD cue can be ‘gated’ by independently manipulating the border delineation indicates that the AD cue is indeed distinct from the border delineation. The fact that the predicted AD percept can be restored by solely better delineating the occluder without changing its flicker indicates that the flicker itself does not affect the accretion-deletion cue. It also indicates the failure of the AD cue in stimuli with flickery surrounds is not due to trivial causes, such as the inability to resolve individual pixels. Together, these findings support the aforementioned computational results, and show that the visual system needs additional information about the occlusion border in order to use the occlusion/disocclusion information.

## Discussion

Our results demonstrate that it is possible, in principle, to unambiguously determine the depth-order of moving objects solely by keeping track of the accretion/deletion of the occluded object. However, we also show empirically that the human brain is unable to do determine depth-order in this fashion, and that the accretion-deletion information by itself is ambiguous to the visual system. The visual system needs additional constraining information about the occlusion border.

### Moonwalk Illusion as Bayesian ‘Explaining Away’

The percepts elicited by our stimuli, including the depth-order effects, can be readily explained as a well-known type of Bayesian inference called ‘explaining away’[20], [21]; also see [12], [14]. Briefly, explaining away refers to a scenario where the stimulus supports two alternative interpretations, either one of which is plausible in the absence of additional constraining evidence. But when the constraining evidence is available, one of the two original interpretations becomes much more plausible. In the present case, the two plausible interpretations of our stimuli are shown in Fig. 1d and 1e, respectively. The AD cue is consistent with only one of the interpretations (Fig. 1d), but our results show that the visual system cannot use this information by itself. The constraining evidence is provided by the segmentation cue, and this additional evidence makes the interpretation in Fig. 1d much more plausible. Thus, when strong enough segmentation information is available, the brain favors the interpretation consistent with the AD cue, using the segmentation information to explain away ambiguities in the incoming AD information.

In the absence of strong enough segmentation cues, the brain is unable to use the AD cue by itself to determine DFM. Hence it chooses the alternative information where the center surface is perceived as translating in the near plane (Fig. 1e). Note that, in the absence of usable evidence that the center is occluded, translation of the center accounts for the dot motion in the center. The perception of the center as near in this case is also aided by the built-in perceptual bias to interpret the shearing created by translating surfaces as nearness cue[15]. In this case, in the absence of strong enough gating information, *i.e*., segmentation cues, the brain chooses an interpretation to account for the remaining available information.

### Implications for the Neural Substrates of DFM

Our results also provide useful constraints on the neural mechanisms by which the brain processes the AD cue. The fact that unlike the Ideal Observer, the visual system cannot use the AD cue by itself to determine DFM in a “bottom-up” fashion, suggests that the extraction of AD information is closely associated with segmentation processes. To the extent that this involves a comparison of the relative velocities of the image elements undergoing accretion/deletion *vs*. the border between the two surfaces[22], [23], [24], the underlying process is, by definition, a second-order motion process[9], [25], [26], [27], [28], [29]. In other words, the AD cue cannot be processed solely by first-order (*i.e*., luminance-based) motion mechanisms; second-order (*i.e.,* non-luminance based) mechanisms must be involved. Our results also predict that the neural mechanisms of AD cue processing will be different when the occluder is stationary *vs*. moving, even when the accretion-deletion information itself is identical between the two conditions. This is because occluder motion typically gives rise to the BF cue, and the motion of the occlusion boundary creates strong segmentation cues.

Our results disprove the conventional view, nearly half a century old, that the AD cue is self-sufficient for the perception of DFM[1], [2], [3], [4], [6], [7], [8], [9]. In doing so, they offer a new perspective of the relative roles of the DFM cues in DFM perception. Contrary to conventional wisdom, the two known DFM cues are not functionally equivalent (*i.e*., not mutually redundant), but instead play different, perhaps complementary, roles in the perception of depth-order from motion *vs*. surface segregation. As noted above, the BF cue pertains to the motion of the occluder, and the AD cue pertains to the motion of the occluded object. The two cues also engage the first *vs*. second-order motion systems differently[1], [2], [3], [4], [6], [7], [8], [9]. Since the two cues tend to co-occur under natural viewing conditions[1], [2], [3], [4], [6], [7], [8], [9], one cue may serve to compensate for the ambiguities in the other.

## Supporting Information

Supporting Information S1 **Contains various inter-related lines of evidence, including the Ideal Observer analysis, that support the findings presented in the main text.**
(DOCX)
