# Supplementary material for: What the ‘Moonwalk’ Illusion Reveals about the Perception of Relative Depth from Motion
Source: PLoS One. 2011 Jun 22;6(6):e20951. doi: 10.1371/journal.pone.0020951 (PMC3120826; doi:10.1371/journal.pone.0020951)
Supplement: Supporting Information S1 — Contains various inter-related lines of evidence, including the Ideal Observer analysis, that support the findings presented in the main text. (DOCX) [file pone.0020951.s001.docx]

**What the ‘Moonwalk’ Illusion Reveals about the Perception of Relative Depth from Motion**

Sarah Kromrey, Evgeniy Bart, and Jay Hegdé

**SUPPORTING INFORMATION**

**_____________________________________**

**Section S1. Using space-time (ST) plots as static 2-D representations of motion stimuli**

| 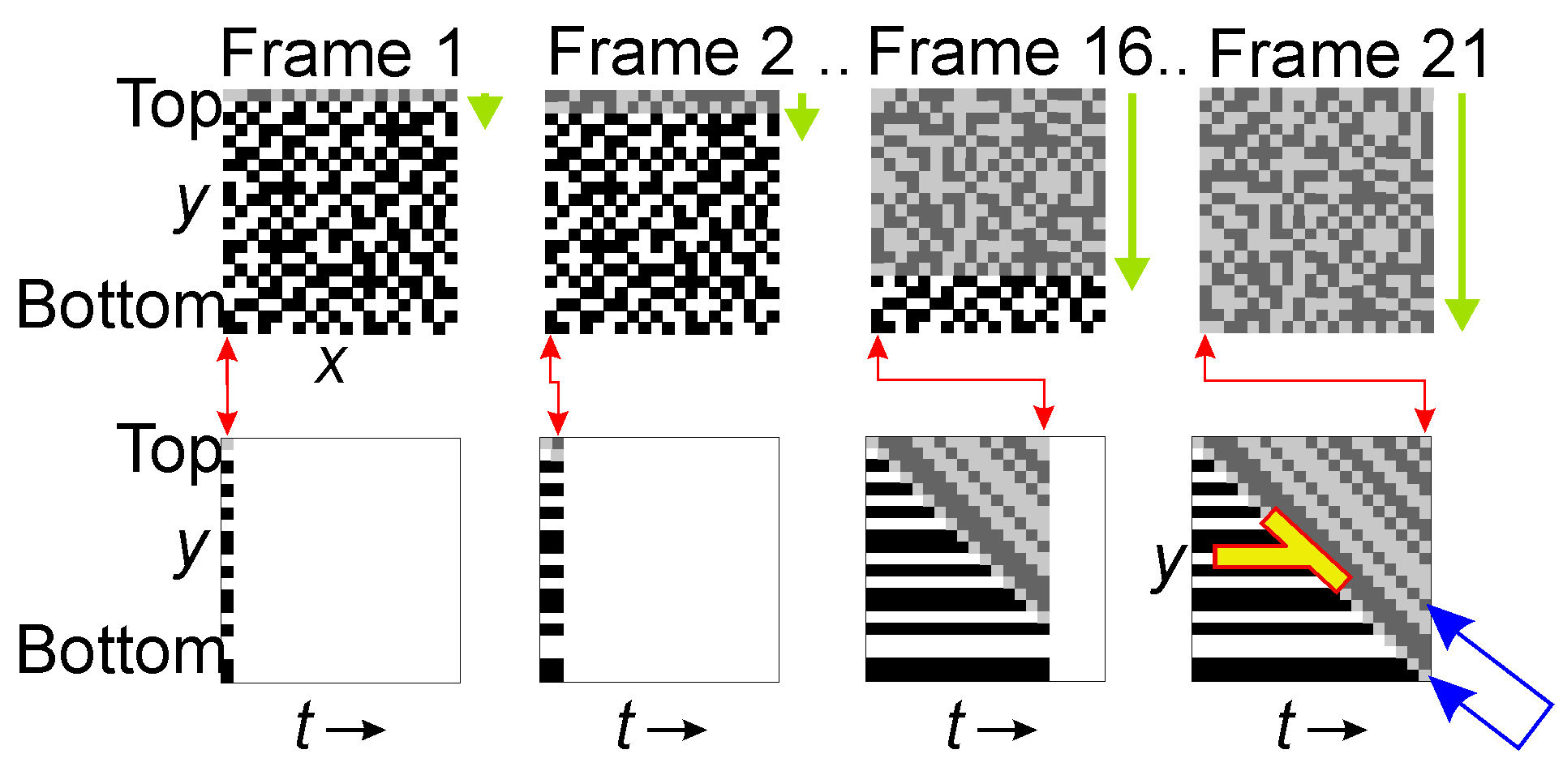  Supplemental Figure S1. Space-time (ST) plots of motion stimuli. |
| --- |

ST plots are often used to represent motion stimuli in a static 2-D form [[1](#_ENREF_1),[2](#_ENREF_2)]. Figure S1 illustrates the construction of ST plots using an exemplar random dot movie with 21 frames. The top row shows four selected frames of the movie; the bottom row shows the corresponding stages of the ST plot construction. The icon at bottom right denotes the finished ST plot.

The ST plot is constructed by concatenating a designated column of pixels from successive frames of the motion stimulus, so that successive columns of the ST plot represent the temporal progression of a given set of pixels in the motion stimulus. For the stimulus shown, the top panel (highlighted in gray) slides downward over the stationary bottom panel in successive frames (green downward arrows). In the present case, the ST plot represents the temporal progression of the far left column of pixels of the movie. However, the choice of which column (or row) to use depends on the user.

When the motion stimulus contains AD and/or BF cues (see main text), the ST plot will show the corresponding diagnostic features. In the present case, the horizontal and oblique lines in the ST plot correspond to stationary and moving textures, respectively. The accretion-deletion (AD) cue is indicated by the termination of the horizontal lines (highlighted by the yellow T) at the boundary (*i.e*., where the two sets of lines meet) between the two panels [[2](#_ENREF_2)]. The BF cue is reflected by the fact that the orientation of the boundary (which denotes the velocity of the boundary) and that of the surface shown in dark gray (which denotes the velocity of this surface) are exactly parallel (paired arrows; also see ref. [[2](#_ENREF_2)]). Note that the BF cue is a property of, and denotes, the near surface, whereas the AD cue denotes the far surface.

**Section S2. Ideal Observer Analysis of DFM**

**S2.1. Notation**

| 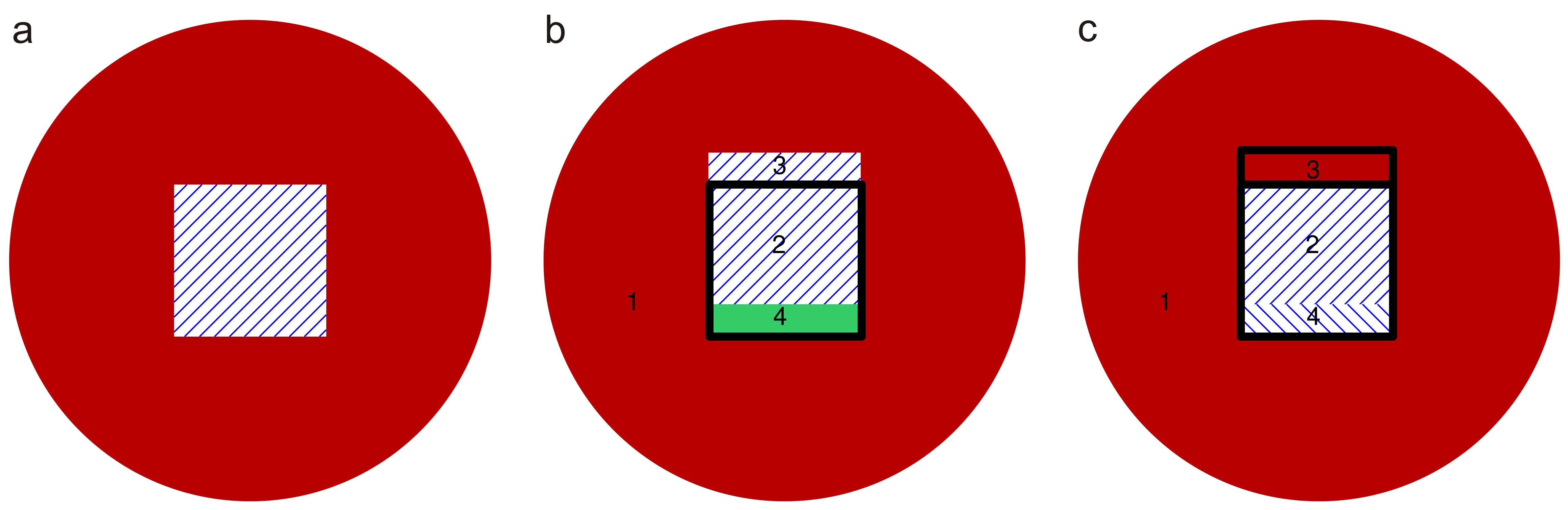  Supplemental Figure S2: Image notation. (**a**) Frame 1 of the motion stimulus. The center and the surround are shown as hatched and red surfaces, respectively. (**b**) Frame 2, in a scenario where the center is near (*i.e*., in front of the surround). (**c**) Frame 2, in a scenario where the center is far (*i.e*., behind the surround). See below for details. |
| --- |

The stimulus consists of a center (hatched) and a surround (red). The center is shown as rectangular in Figure S2 for clarity; however, note that the definitions and the derivation do not change if the center has arbitrary shape. Experimentally, the depth-order percepts elicited by rectangular and irregular-shaped stimuli are statistically indistinguishable (see Section S4 below).

Panel (a) shows the first frame of the sequence. Panel (b) shows the second frame of the sequence when the center is near (in front of the surround). The center has moved up. The black square is the ‘old’ footprint of the center in frame 1. The part of the center that has moved out of the old footprint is referred to as area 3. In area 3, part of the surround is occluded. The part of the center that is still inside its old footprint is area 2. The part within the old footprint which the center revealed after moving is area 4 (small green rectangle). Note that area 4 was not visible before. The remainder of the image is called area 1. Panel (c) shows the second frame of the sequence if the center is far (behind the surround). The center has moved up. The black square is the footprint of the center from frame 1. Since the center is behind, the footprint of the center remains the same in frame 2. Area 3 (also enclosed by a black frame) is the same region in the image corresponding to area 3 in panel (b). It is in red to indicate that surround is still visible in that area. The part of the center that is still visible is area 2. This is the same image region as area 2 in panel (b), both in terms of location within the image and the visual content of the region. The new part of the center that became visible due to motion is area 4. This is the same location in the image as area 4 in panel (b). It is hatched to indicate that it belongs to the center. The hatching is in a different direction to indicate that this is a previously invisible part of the center. Everything else is area 1, and it is the same as area 1 in (b), in terms of both location within the image and the visual content. To summarize, areas 1, 2, 3, and 4 each correspond to the same locations in panels (b) and (c). So we will also use these locations when referring to frame 1 in panel (a). The visual content of these areas may be different in different images.

**S2.2. Decision-making**

We emphasize that the purpose of the following analyses is solely to demonstrate that it is possible, from a strictly information processing viewpoint, to extract DFM information from accretion-information alone. Since this issue is independent of whether the visual system can actually *utilize* such DFM information (if any), the following analysis does not attempt to show (or, for that matter, claim) that the underlying information processing steps are biologically feasible.

All stimuli are generated from the model where the center is far (*i.e.,* behind the surround). So if frame 1 (denoted $I^{1}$) is as in panel (a), then frame 2 (denoted $I^{2}$) is as in panel (c). The observer must decide between two models: the center is far (model denoted by *F*) and the center is near (model denoted by *N*). For this, we need to compare *p*($I^{1}$, $I^{2}$|*F*) and *p*($I^{1}$, $I^{2}$|*N*).

Now,

*p*(* *^^ * *^^|*F*) = *p*(* *^^*F*)*p*(* *^^* *^^, *F*), (1)

and

*p*(* *^^ * *^^|*N*) = *p*(* *^^*N*)*p*(* *^^* *^^, *N*). (2)

*p*($I^{1}$|*F*) and *p*($I^{1}$|*N*) are the prior probabilities of the first frame, computed without seeing any other images. These do not depend on the occlusion model (near or far), but only on the image model (i.e., what we think images look like). Therefore it is natural to assume *p*($I^{1}$|*F*) = *p*($I^{1}$|*N*). Note that this is the case even when priors for the center and the surround are different, because when only one frame (the frame $I^{1}$) is considered, the amount of center pixels is the same for the two models, and similarly for the surround pixels. Under this assumption, we only need to compare *p*($I^{2}$|$I^{1}$, *F*) and *p*($I^{2}$|$I^{1}$, *N*) to decide on the *F* *vs*. *N* model. Next, we propose an ideal observer model for how this comparison is performed.

**S2.3. Ideal Observer model**

We assume that the center location, shape, and velocity are known. Similar assumptions are standard in comparable analyses [[3](#_ENREF_3)]. The rationale is that these can be very accurately estimated after watching a few frames of the stimulus. One exception is the location of area 3, which may be difficult to be estimated precisely due to flicker.

We also use the ‘brightness constancy’ model of image appearance for the center and a flicker model for the surround. These models are detailed below.

For the center, the conventional brightness constancy assumption [[4](#_ENREF_4),[5](#_ENREF_5),[6](#_ENREF_6)] states that for two corresponding pixels, *i*_1_ in frame 1 and *i*_2_ in frame 2,

$I_{i_{2}}^{2}$ — $I_{i_{1}}^{1}$ ~ *N*(0, σ), (3)

where $I_{i}^{f}$ is the grey value of frame *f* at pixel *i*. That is, we assume that the noise is Gaussian with zero mean and standard deviation σ. Usually σ is assumed to be small. Since center does not flicker in our stimuli, this is a reasonable assumption.

Since the image is binary in our case, we modify the brightness constancy model as follows: the pixel flips with probability *p_c_* (which is a new parameter of the model replacing σ), and remains unchanged with probability 1 − *p_c_* . The model becomes:

|$I_{i_{2}}^{2}$ — $I_{i_{1}}^{1}$| ~ ${\{}_{1 with probability p_{c}}^{0 with probability 1-p_{c}}$ (4)

Brightness constancy would not model the surround well. The reason is that the surround flickers, and therefore changes in appearance are expected. It is therefore desirable to replace brightness constancy with a more accurate model of surround appearance. Here, we assume a two-stage model for flicker. In the first stage, the model observes the stimulus and estimates from it the flicker probability *r*. In the second stage, it uses the estimated distribution of *r* given the data observed so far to evaluate the likelihood of the surround from subsequent frames. This appearance model monitors the number (or area) of pixels that changed between frames (biologically, this could be done using motion detectors or change detectors), and penalizes deviations of the number of pixels that actually flipped from the expected value *r*.

Suppose the model has observed the stimulus for several frames. Denote the total number of observed pixels by $N_{0}$ (this is the number of pixels in area 1 times the number of frames). Of these, denote the number of pixels that flipped by $N_{0}^{f}$. The number of pixels that didn’t flip is then $N_{0}^{c}=N_{0}-N_{0}^{f}$. Assuming a uniform prior on *r*, the posterior is $\mathrm{Beta}(r|N_{0}^{f}+1, N_{0}^{c}+1)$, *i.e.*, the Beta distribution with parameters $\alpha=N_{0}^{f}+1$ and $\beta=N_{0}^{c}+1$.

Next, the model observes area 3 for two consecutive frames. Denote the total number of pixels in area 3 by $N_{3}$. Denote the number of pixels that have flipped by $N_{3}^{f}$, and the number of pixels that didn’t flip by $N_{3}^{c}=N_{3}-N_{3}^{f}$. We assume $N_{3}^{f}$ is distributed binomially with the probability of flip *r*, where *r* itself is distributed according to $\mathrm{Beta}(r|N_{0}^{f}+1, N_{0}^{c}+1)$ (the posterior estimated in the previous step). The surround model $p(N_{3}^{f})$ is then obtained by integrating out the parameter *r* from the joint distribution $p(N_{3}^{f}, r)$. It is straightforward to show that

$p\left( N_{3}^{f} \right)= \frac{B(N_{0}^{f}+N_{3}^{f}+1, N_{0}^{c}+N_{3}^{c}+1)}{B(N_{0}^{f}+1, N_{0}^{c}+1)} \left( \begin{matrix} N_{3} \\ N_{3}^{f} \end{matrix} \right).$ (5)

Note that this takes into account the uncertainty in estimating *r* from observations.

Again, note that the surround appearance model models the flicker rather than the image appearance directly. This is because random changes in the surround appearance are expected, and trying to predict the exact pixel-wise appearance of the subsequent frame is therefore impossible and useless.

Note that although the pixels in area 3 are independent given *r*, integrating *r* out introduces a dependency between these pixels and we no longer can model them as independent. Intuitively, the reason is that evaluating the flicker is best carried out by pooling over multiple pixels. We do, however, assume that the four image areas (Figure S2) are treated independently. The reason is that flicker can be computed from a small image patch, much larger than a single pixel, but much smaller than any of the four image areas. Such local patches can be treated independently, and due to their small size they are unlikely to span multiple image areas.

Under this assumption,

*p*(* *^^* *^^, *F*) = *p*($I_{[area 1]}^{2}$|$I^{1}$, *F*) **^.^** *p*($I_{[area 2]}^{2}$|$I^{1}$, *F*) **^.^**

*p*($I_{[area 3]}^{2}$|$I^{1}$, *F*) **^.^** *p*($I_{[area 4]}^{2}$|$I^{1}$, *F*) (6)

and

*p*(* *^^* *^^, *N*) = *p*($I_{[area 1]}^{2}$|$I^{1}$, *N*) **^.^** *p*($I_{[area 2]}^{2}$|$I^{1}$, *N*) **^.^**

*p*($I_{[area 3]}^{2}$|$I^{1}$, *N*) **^.^** *p*($I_{[area 4]}^{2}$|$I^{1}$, *N*) (7)

Now, pixels in area 1 belong to the surround under both models *F* and *N*. Therefore, *p*($I_{[area 1]}^{2}$|$I^{1}$, *F*) = *p*($I_{[area 1]}^{2}$|$I^{1}$, *N*), and pixels in area 1 can be ignored in the comparison.

Pixels in area 2 belong to the moving center in both models *F* and *N*. Moreover, since the center moves with the same velocity in *F* and *N*, the pixels in area 2 have the same corresponding region in frame 1 for both models *F* and *N*. Therefore, *p*($I_{[area 2]}^{2}$|$I^{1}$, *F*) = *p*($I_{[area 2]}^{2}$|$I^{1}$, *N*), and area 2 can be ignored in the comparison as well. Finally, pixels in area 4 belong to the previously invisible part of the center in model *F*. These pixels belong to the previously invisible part of the surround in model *N*. The size and location of this area is the same in models *F* and *N* (because the direction and speed are the same). So they do not depend on the motion model, but rather only on the prior information about image appearance. More specifically, in *F*, this is the prior on center appearance, and in *N*, this is the prior on surround appearance. In our case, the two priors are the same (random black-and-white pixel noise). More generally, whenever we talk about kinetic edges, the two priors must be the same—otherwise the edge between center and surround will not be purely kinetic. Therefore, *p*($I_{[area 4]}^{2}$|$I^{1}$, *F*) = *p*($I_{[area 4]}^{2}$|$I^{1}$, *N*), and area 4 can be ignored in this comparison as well (see below).

Note that in depth model *F*, area 4 represents the region of the image where the center pixels are disoccluded over successive frames. Previous studies have shown that human observers can use disocclusion to determine depth-order, just as they can use occlusion [[7](#_ENREF_7),[8](#_ENREF_8),[9](#_ENREF_9)]. It should be noted that, while our model ignores the disocclusion information (in area 4) for the sake of simplicity, it is straightforward to expand the model to incorporate this information in one or both of the following two ways. First, if the priors on center and surround appearance are different (as in the window shade example or in the experiments where the center and surround contrast was different, Fig. 4), then *p*($I_{[area 4]}^{2}$|$I^{1}$, *F*) ≠ *p*($I_{[area 4]}^{2}$|$I^{1}$, *N*), and area 4 will become informative for the decision. Second, note that the current model considers only two consecutive frames. If integrating over multiple frames is added, area 4 might again become useful for the comparison. Note also that the goal of the current analysis is to show that depth order information is still present in the stimuli, rather than to model the human decision process. Therefore modeling these additional decision factors is not necessary, since they only add, but do not reduce, the amount of information present in the stimuli. It is of interest that the subjects ignore this multi-frame information as well (as is evident from the illusion), but this is not the focus of the current paper.

In summary, to decide between *F* and *N* in the current model, we only need to compare *p*($I_{[area 3]}^{2}$|$I^{1}$, *F*) to *p*($I_{[area 3]}^{2}$|$I^{1}$, *N*), and the log-likelihood ratio is

*L* ($p_{c}$) = log $\frac{p(I_{\left[ area 3 \right]}^{2}|I^{1}, F)}{p(I_{\left[ area 3 \right]}^{2}|I^{1}, N)}$ (8)

Next, we compute this log-likelihood ratio for our stimuli. Note that the actual stimuli are always from the model *F*. Therefore, all pixels in area 3 belong to the surround. When the surround is dynamic, the pixels may flip with probability *f*. Therefore, for all pixels *i* in area 3, $I_{i}^{2}$ = $I_{i}^{1}$ with probability 1 − *f* and $I_{i}^{2}$ ≠ $I_{i}^{1}$ otherwise.

We compute the probabilities of these stimuli under models *F* and *N*.

For *F*, the model prediction is that area 3 corresponds to the same surround region in both frames. Therefore,

*p*($I_{[area 3]}^{2}$|$I^{1}$, *F*) = $p(N_{3}^{f})$ (9)

$=\frac{B(N_{0}^{f}+N_{3}^{f}+1, N_{0}^{c}+N_{3}^{c}+1)}{B(N_{0}^{f}+1, N_{0}^{c}+1)} \left( \begin{matrix} N_{3} \\ N_{3}^{f} \end{matrix} \right)$ (10)

In our stimuli, the fraction of pixels that flip is *f*. Therefore, $N_{3}^{f}=fN_{3}$, and $N_{0}^{f}=fN_{0}$. It is reasonable to assume that $N_{3}\ll N_{0}$. This is because $N_{0}$ consists of many pixels observed over multiple frames, whereas $N_{3}$ includes only pixels in the (relatively small) area 3 over one pair of frames. In this case, and using the Stirling approximation, we obtain

$p\left( I_{\left[ area 3 \right]}^{2} | I^{1}, F \right)\approx\frac{1}{\sqrt{2\pi N_{3}f(1-f)}}$.

This approximation applies to most values of *f* except those close to 0 (or to 1). The reason is that when $f\approx0$, $N_{0}$ approaches 0 and the Stirling approximation becomes inaccurate.

The model *N* predicts that pixel *i* in area 3 will correspond to some pixel on the center. Denote the value of this center pixel by *C_i_*. That is, *C­_i_* is the value of that pixel on the center which would correspond to pixel *i* in the second frame given the center’s location and velocity. Therefore,

*p*($I_{\left[ area 3 \right]}^{2}$|$I^{1}$, *N*) = ∏*_i_*_area 3_ *p*($I_{i}^{2}$ = *C_i_*) = $p_{c}^{N_{\mathrm{diff}}}$ ${(1-p_{c})}^{N_{\mathrm{same}}}$(11)

Here *N*_same_ is the number of pixels that agree with the prediction (*i. e*., those for which $I_{i}^{2}$ = *C­_i_*), and *N*_diff_ is the number of pixels that are different from the prediction. In our case, both the center and the surround consist of random dots, 50% black and 50% white. Therefore, *p*($I_{i}^{2}$ = *C­_i_*) = 0.5, and *N*_same_ ≃ *N*_diff_ ≃ $N_{3}/2$.

Therefore, the log-likelihood ratio *L* is

$$L\left( p_{c} \right)= \log p\left( I_{\left[ area 3 \right]}^{2} | I^{1}, F \right)-\log p_{c}^{\frac{N_{3}}{2}}\left( 1-p_{c} \right)^{\frac{N_{3}}{2}}=$$

$-\frac{1}{2}log (2\pi N_{3})-\frac{1}{2}\log f(1-f)- \frac{N_{3}}{2}\log p_{c}\left( 1-p_{c} \right).$ (12)

Note that this equation is dominated by the last term, which is proportional to $N_{3}$. The first term is a constant proportional to $\log N_{3}$. The second term is small and nearly constant. More precisely, as *f* changes from 0.5 to 0.01, the second term changes from 0.6 to 2.4. For comparison, if $p_{c}=0.01$ and $N_{3}=100$, then the third term is equal to 231. Therefore,

$$L\left( p_{c} \right)\approx- \frac{N_{3}}{2}\log p_{c}\left( 1-p_{c} \right)=const.$$

The Ideal Observer judges the center to be far if *L* > 0, and judges the center to be near if *L* < 0.

For reasonable (*i. e*., small) values of *p_c_*, *L* is positive. Therefore, the Ideal Observer judges the center to be far for all values of flicker *f*. Note also that the expression is independent of *f*. This means that the confidence of the Ideal Observer is unaffected by flicker.

**Section S3. Perception of AD stimuli as a function of eccentricity**

| 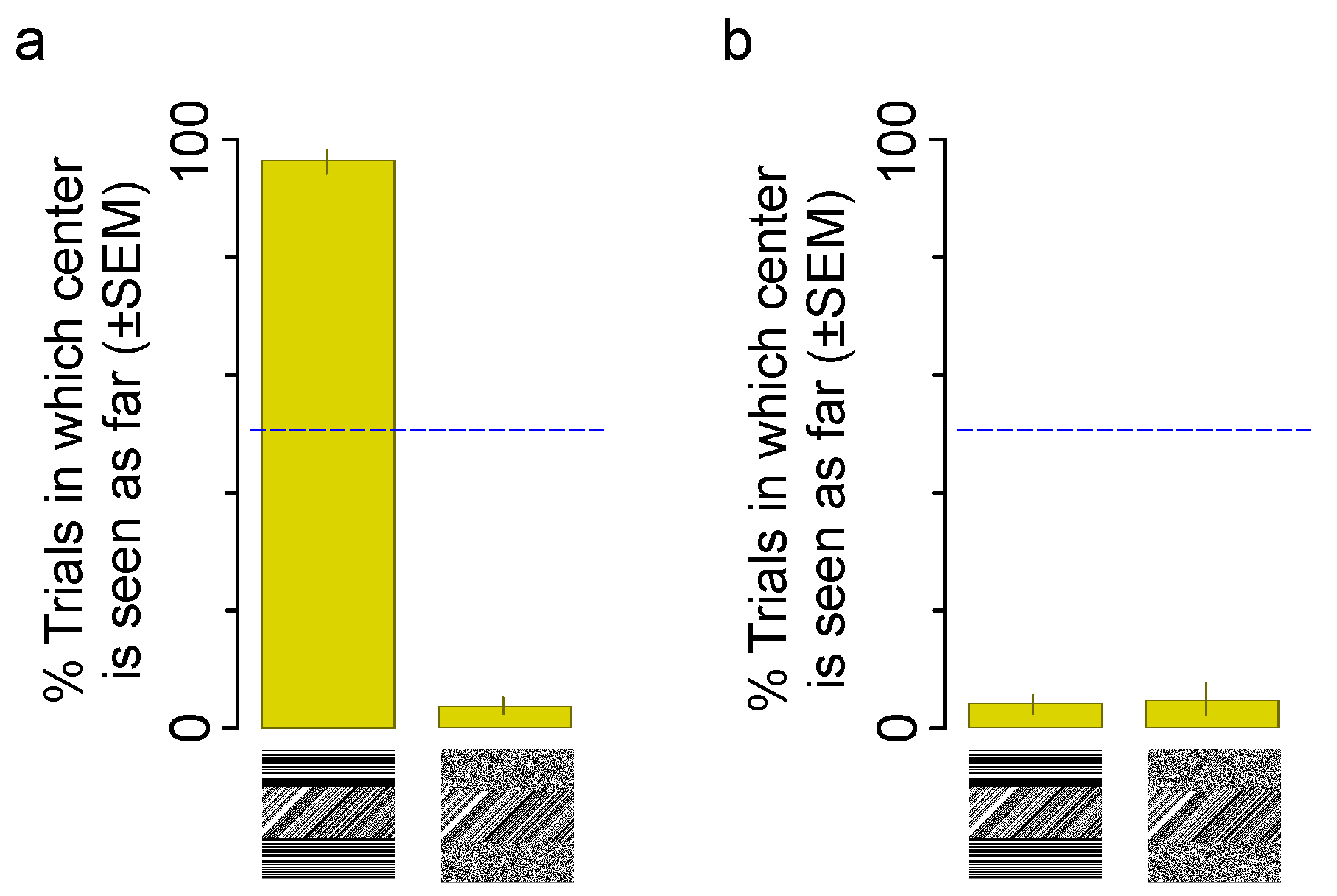  Supplemental Figure S3. Depth-order perception of our AD stimuli as a function of stimulus eccentricity. (**a,b**) DFM percepts elicited by the same pair of stimuli when they are viewed foveally (**a**) and parafoveally at an eccentricity of 5^O^ (**b**). |
| --- |

Our preliminary results (not shown) indicated that when the stimuli are viewed parafoveally, depth-order is perceived incorrectly even more often than with foveal viewing. Here we document this effect quantitatively and argue that this does not weaken the arguments presented in the main text.

Panel a in Fig. S3 replicates the effect shown in Fig. 2 of the main text, in which the stimuli were viewed foveally. As expected, the depth-order reversed when the surround is static *vs*. flickery (Fig. S3a left *vs*. right, respectively). However, when the same pair of stimuli is viewed parafoveally, the depth-order does not reverse for flickery surrounds, *i.e.,* the center is perceived as near regardless of whether the surround is stationary or flickery (Fig. S3b left and right bars, respectively).

It is important to emphasize that this lack of depth-order reversal does not in any way diminish or disprove our arguments regarding the insufficiency of the AD cue. Note that with parafoveal viewing, *both* stimuli elicit percepts that are *opposite* of the percept predicted by the AD cue. Thus, if anything, this strengthens our arguments about the insufficiency of the AD cue by presenting another case in which the AD cue fails to dictate the DFM percept.

It is noteworthy that the stimuli in Fig. S3b do not elicit chance-level performance, but consistently elicit near percepts instead. We show below (Section S4) evidence that indicates that a possible explanation for this effect is that the visual system has a perceptual bias to interpret shear as a nearness cue.

**Section S4. Biasing effects of motion shear**

| 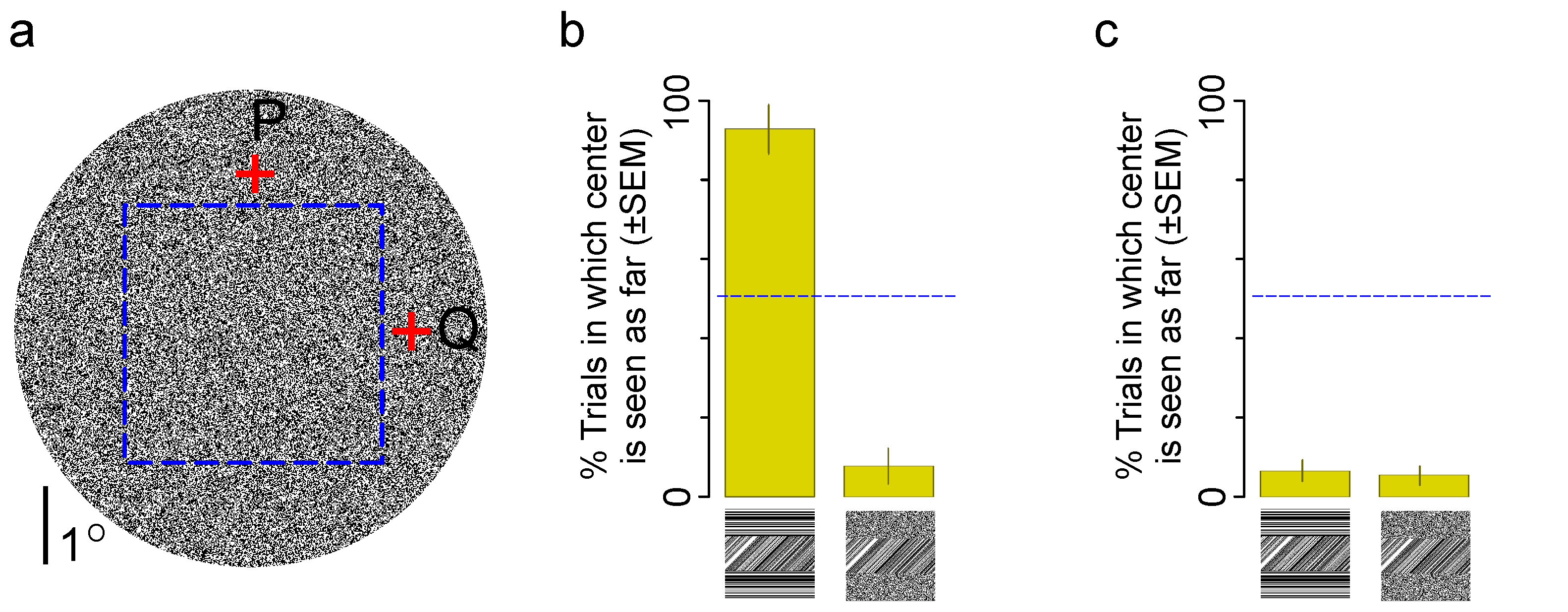  Supplemental Figure S4. Depth-order perception of our AD stimuli as a function of the position of fixation relative to the AD border *vs*. shear border. See text for details. |
| --- |

In this experiment, the center was a square. When the center dots move vertically (similar results were obtained for other directions of movement), the top and bottom sides of the center represent the AD border, or the border at which the dots undergo accretion/deletion. Similarly, the vertical sides represent the shear border, along which the center dots are in a shear motion relative to the surround dots.

When the subjects fixated along the horizontal border (fixation position P), the results (Fig. S4b) essentially replicated those in Fig. 2 of the main text. Note, incidentally, that this also indicates that the square stimulus used in the Ideal Observer analysis can elicit the relevant results.

When the subjects fixated along the vertical border (fixation position Q), which also had the effect of increasing the eccentricity of the AD border, the subjects reliably reported near percepts (Fig. S4c).

The above effects can also be elicited by fixating at the center of the stimulus and attending to horizontal *vs*. vertical borders (data not shown). (Also see Demo Movie 2.) These results are consistent with the results of Royden and colleagues who have shown similar effects when the shear border is made more prominent than the AD border [[10](#_ENREF_10)].

It is important to emphasize that shear is solely a perceptual bias, and is not a stimulus-based DFM cue like the AD and BF cue are. This is because the particular type of shear that elicits the depth-order bias in this case, in which one surface is stationary while the other is moving, can just as easily result when the moving surface is nearer or farther than the stationary surface.

Taken together, these considerations suggest that when the stimulus-driven DFM evidence (in this case, the AD cue) is weak, instead of interpreting the stimulus randomly (*i.e*., at chance levels), the brain relies more on its perceptual biases based on the shear cue. Thus, the DFM percept at any given viewing location represents the relative balance between the strengths of stimulus-driven, or ‘bottom-up’ cues *vs*. ‘top-down’ signals such as the near bias resulting from shearing motion.

**Section S5. Flickering occluder does not necessarily diminish the AD cue, and a flickering surface is not necessarily reported as the far surface.**

| 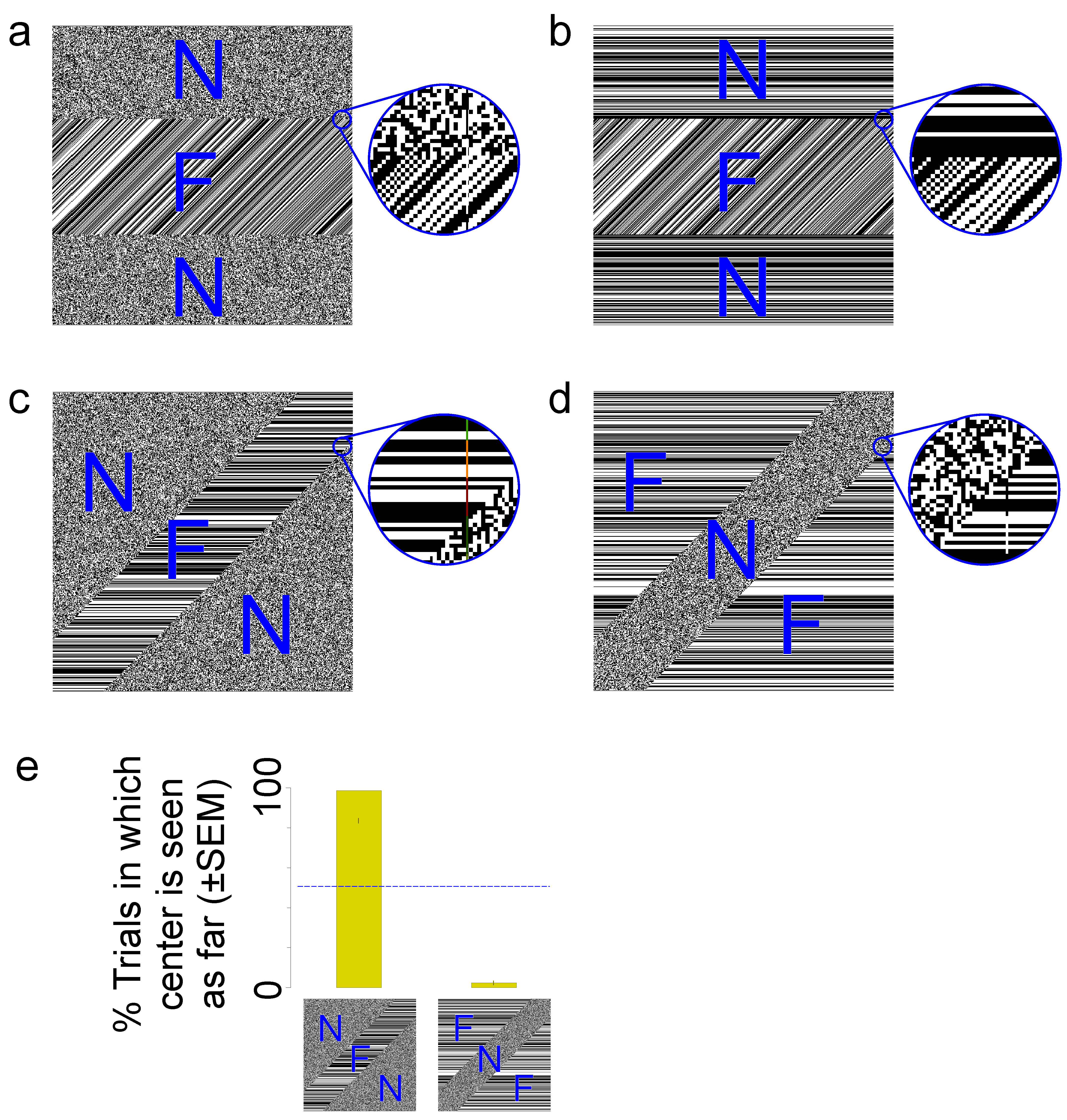  Supplemental Figure S5. AD cue with or without the motion of the AD border. See text for details. |
| --- |

As noted in the main text, one potential concern about our depth-order illusion is that introducing the flicker in the surround may somehow affect the accretion-deletion information in the center. Using conventional optical flow techniques, we have shown in Fig. 3 of the main text that the flicker in the surround does not affect the available accretion-deletion information in the center in our stimuli. Here we provide psychophysical evidence to further support this view.

Figure S5 (a) and (b) are ST plots of the two main stimuli used in our study. Both represent a static occluding surface and a moving occluded surface. The corresponding insets show that the termination of motion trajectories, which constitute the AD cue ([[1](#_ENREF_1),[2](#_ENREF_2)]; also see Fig. S1 above), are unaffected by the flicker.

Previous studies have shown that when the occluder moves, the AD cue can elicit the predicted percepts, *even when the occluder is flickery* [[1](#_ENREF_1),[2](#_ENREF_2)]. Figure S5 (c) and (d) both show such stimuli with flickering, but moving, occluders. The sole DFM in these stimuli is the AD cue (see Fig. 2 of ref. [[1](#_ENREF_1),[2](#_ENREF_2)]).

Note that the motion trajectory terminators in these stimuli are directly comparable to those in panels a and b (insets). Thus, if flickering occluder affects the AD cue in the occluded object, then the actual percepts should be different than those predicted by the AD cue (blue letters; N=near, F=far). Subjects reported the depth-order of the middle surface (which represents the occluded surface in Fig. S5c and the occluder in Fig. S5d). However, the reported percepts were entirely consistent with the predicted percepts. Taken together, these results indicate that the flickering occluder by itself does not diminish the perceptual efficacy of the AD information. In other words, the motion terminators do remain interpretable as AD cue. Rather, what diminishes the AD cue when the occluder is static in our stimuli is the absence of strong cues about the AD border. When the surround is static and flickery, the border is not clearly distinguishable (which can be ascertained from Demo Movie 2). However, when the occluder moves, even though it remains flickery, it clearly delineates the border between the occluder and the occluded surface. Thus, information unrelated to accretion-deletion *per se*, namely segmentation information that delineates the occluded surface from the occluder, is needed to constrain the AD cue. This also helps explain when the border between the two surfaces is delineated by static segmentation cues such as contrast, color and luminance, it restores the DFM percepts predicted by the AD percept (see main text).

The results shown in Fig. S5 also address a potential concern about the Moonwalk illusion. Recall that in the illusion, the perceived nearness of the center is closely linked with the flickering surround, in that the subjects reported the center as near when the surround was flickering, and center as far when the surround was not flickering. One possible confounding explanation for this effect is that the subjects spuriously associated the surround flicker (or the presence of flicker anywhere in the stimulus) with the nearness of the center, and reported their depth-order percepts accordingly. This confound is highly unlikely, for three reasons. First, the depth-order reports of the subjects are self-evidently valid, in that the stimuli do elicit the corresponding reported percepts, as can be verified from Demo Movies 1 and 2. Second, subjects had no reason to form the aforementioned spurious associations, since they were told to report what they saw, and that there was no ‘correct’ percept *per se*. Thus, for the above confound to be valid, all subjects would have had to ignore the instructions and formed the exact same spurious association. Third, as shown in Fig. S5, the same subjects also reported the flickering surface as the near surface in other stimuli that contained a flickering surface, indicating that the subjects did not necessarily report flickering surfaces as far, or report the center as near whenever there was flicker anywhere in the stimulus. Taken together, these considerations indicate that the Moonwalk percept is not attributable to this confound.

**SUPPLEMENTAL REFERENCES**

1. Adelson EH, Bergen JR (1985) Spatiotemporal energy models for the perception of motion. J Opt Soc Am A 2: 284-299.

2. Hegdé J, Albright TD, Stoner GR (2004) Second-order motion conveys depth-order information. J Vis 4: 838-842.

3. Weiss Y, Simoncelli EP, Edelson EH (2002) Motion illusions as optimal percepts. Nat Neurosci 5: 598-604.

4. Wallach H (1948) Brightness constancy and the nature of achromatic colors. J Exp Psychol 38: 310-324.

5. Leibowitz H, Myers NA, Chinetti P (1955) The role of simultaneous contrast in brightness constancy. J Exp Psychol 50: 15-18.

6. Ullman S (1979) The interpretation of Visual Motion. Cambridge, MA: MIT Press.

7. Gibson JJ, Kaplan GA, Reynolds HEN, Wheeler K (1969) The change from visible to invisible: A study of optical transitions. Percept Psychophys 5: 113-116.

8. Kaplan GA (1969) Kinetic disruption of optical texture: the perception of depth at an edge. Perception and Psychophysics 6: 193-198.

9. Howard IP, Rogers BJ (2002) Seeing in Depth. Vol. 2. Depth Perception: I. Porteous, Toronto.

10. Royden CS, Baker JF, Allman J (1988) Perceptions of depth elicited by occluded and shearing motions of random dots. Perception 17: 289-296.
